# Pyemia of Many Years’ Standing from Concealed Abscess, Caused by a Wisdom Tooth

**Published:** 1883-01

**Authors:** 


### Pyemia of Many Years' Standing, from Concealed Abscess, Caused by a Wisdom Tooth.

[Read before the Cincinnati Dental Society, Nov. 14, 1882, By R. J. Porre, D D.S.]

The history of the case, in some of its prominent features, is as follows.

J. McD., in the latter part of June, 1882, consulted a physician of this city for treatment. His age is fifty-two years, of rather light build, nervo-sanguine temperament, very muscular, and in his early life somewhat noted as an athlete, inheriting a powerful constitution and free from hereditary taint. From his youth he had been employed in active business. Had served from the beginning to the end of the late sectional war. Was proprietor and master of a steamboat for four years, having to abandon it on account of his health. Then, in a short time, he was appointed to an important office under the Government, which he held for eight years.

For thirty years he had been a great sufferer, both mentally and physically, and was often forced to take to his room and bed for weeks and months at a time, for treatment and recuperation. And that at *no time* during that period can he recollect that he was free from pain in some part of his body. He recalls that his affliction began with inflammation and swelling on the right side of his face and head, to the extent of closing his jaws and forcing him to liquid diet for a week or so, being the victim, meanwhile, of intense suffering. In a short time after the swelling subsided, tumors or boils appeared on different parts of his body, some of them secreting and discharging surprising quantities of pus, and that they have continued in an uninterrupted series to form, either singly or in groups of six or eight, furuncular in class, invariably containing abnormally large cores, which at proper time would be removed, leaving receptacles for the secretion of pus or a sanious fluid which continued to discharge for months before subsiding, and then only to give place to others on other parts of the body, of like character. His hands and feet were constantly involved with an itching and burning eruption, or were a mass of boils and degenerated sloughing tissue, rendering him utterly helpless for many weeks at a time, and exciting, during some attacks, alarming apprehension that he would lose one or both hands, or one or both feet, or portions of them. The throat and vocal organs were frequently so implicated that he was forced to communicate his wishes in a whisper or in writing. Respiration was at periods, difficult and painful, from spasmodic contraction of the diaphragm and muscles of the throat. Bowels almost constantly, for twenty or twenty-five years, so constipated and so seriously complicated with spasmodic constrictions (notably of the pyloric orifice and duodenum, producing the sensation of a plug as he described it,) when, after several days, sudden relaxations would give relief. It was necessary that daily resort be had to artificial aid in the evacuation of the bowel, either in the use of purgatives, injections, or the bougie; and very often all of them utterly failed to evacuate the fecal accumulations under persistent effort for days. During the same time to effect urination, the catheter was daily required, or the bougie to dilate a most painful and obstinate spasmodic constriction of the urethra. Phenomenally profuse and consequently exhausting night sweats from time to time have been persistently combatted by all approved remedies without avail, or with but slight mitigation. Fevers varied in intensity and duration according to the severity of the complications and constitutional conditions, at times intermittant for weeks. A fact I will here mention, as important in reporting this most extraordinary case, is that from the first attack these pains were severe and lancinating, always originated and irradiated from the right side of the inferior maxilla, and that like electric shocks, involved not only the nerves and tissues in that side of the face, head and neck, and sent stiletto-like thrusts through the diaphragm, the bowels, the bladder, the limbs, hands and feet, and with most poignant severity always pierced the most inflamed and tender lesions. This should have led to an investigation of the jaw for the cause, but it did not.

The patient in private life, and while in the army, employed in the treatment of his case, some thirty odd of the most eminent and skilled physicians and surgeons, who had charge for longer or shorter periods, and each in turn diagnosed his symptoms pathognomonic of Syphilis, and, consequently, he was put upon and kept under a vigorous mercurial and potassa iodide course of treatment for thirty years, with but trivial interruption, and has never been benefitted, except, perhaps, when under a temporary course of tonics to place him in condition to resume the regular course again.

On the 30th of August last the patient was sent to me to examine the teeth of the right side, for the cause of the then unusually intense and quite unendurable pain. His physician, while treating his throat, had pressed the gum in the region of the wisdom tooth on the affected side, and accidentally discovered the exudation of a small quantity of pus, and naturally surmised that it was an indication of a lesion that was, perhaps, the cause of his pain. Although I had known the patient for fifteen years in another city, and had knowledge that he was afflicted with boils and eruptions, it was at this visit that I first obtained a limited history of his case. Upon examination of his mouth I discovered no appearance of abscess or inflammation, but observed the imprint of the teeth on the cheek, evidencing atony of the tissues. Pressure upon the outside of the cheek showed tenderness and a slight fullness opposite the wisdom tooth, or location of the wisdom tooth, and base of the ramus, and extending back and down to the angle of the jaw. I saw no wisdom tooth, but upon percussion found slight tenderness in the second molar, and while testing for caries in the crown and drawing the point of an instrument over the posterior proximal surface, felt what proved to be a partially erupted wisdom tooth. By the aid of a mouth glass I discovered that not more than two lines of the anterior grinding surface was exposed to view. It yielded under pressure, showing its detachment from the alveolus by absorption, and the gush of sanious fluid unmistakably revealed the cause of absorption. At once I declared my conviction to the patient, that the fountain head of all his physical wretchedness and torture was unmistakably discovered, and from that hour his treatment for syphilis, which he was then under, should be changed to that of anti-pyemic; and I requested him to so advise his physician. The tooth was immediately removed from its pocket-like investment. The root, as anticipated, was almost entirely absorbed; as the operation had been somewhat painful, I desisted from further examination and syringed the abscess and dressed with a pledget of cotton saturated in dilute carbolic acid and tinct. eucalyptus, and dismissed him with a request to call next day to have the sack treated and again dressed. Being greatly debilitated, and suffering with an unusually severe series of spells with his constrictions, crampings of the diaphragm, etc., exhausting night sweats, in a word, all of the complications, as he described it, had concentrated in force and set upon him at once with relentless fury, to annihilate him. So he did not again call until August 11th. I found the soft tissue socket open as upon the day I removed the tooth. After syringing I introduced a curved pointed steel instrument, to test the condition of the alveolus, and not only found it carious, but found a section of the bone involved from opposite the posterior line of the second molar back and down quite to the extreme angle of the jaw. Puncturing through carious bone as I proceeded to the depth of one and a half to two lines to the solid bone. In my survey for the limits of the area diseased towards the lower margin of the jaw, most unfortunately I punctured or wounded the inferior maxillary nerve. It was evidently dissected as it were, by action of disease from its investment, and lay isolated and exposed to the point of my instrument. The application of a lotion composed of chloroform, tinct. aconiti, morphine, spts camphora, and carbolic acid, soon gave relief. I did not venture a possible repetition of the infliction. I told the patient that before relief could be promised the carious surface must be removed, and that there were two methods of accomplishing it; one was to burr it off with the dental engine, the other was to destroy it with sulphuric acid. The first would require the extraction of the second molar, to which he protested if it could be avoided, as the organ was sound, so I readily decided upon the other, as I preferred it, for having tried it under like circumstances in the destruction of carious and necrosed tissue, and proven it more efficient and satisfactory than any methods with instruments, consequently I rolled a piece of linen lint (cotton will not answer, as it dissolves almost instantly)

into a ball the size of a green pea, and after having absorbed fluids from the sac, took the ball with curved pliers and saturated it in pure sulphuric acid and applied well down and over the carious surface. For an instant or two after application no unpleasant sensation was felt. I was anxiously watching for results, when like a thunder bolt the inferior maxillary nerve yielded to the mastery of the powerful agent, and sent a thrill of agony through every tissue and fibre of the body. But a prompt application of the obtundent again (and forever in that locality it was hoped) gave relief in a very few moments. There was, of course, destruction of the soft tissues to a limited extent. This was desirable. Not only the destruction of the carious bone was required to produce a cure, but healthy action in the connecting tissue was also indispensably required, and nothing locally could accomplish it more effectually or satisfactorily than through the escharotic, and stimulating effect of the agent used. It will be recollected that there was marked degeneration or atony in the muscular tissues of this locality, but there was no lack of nerve sensibility or reaction, rather a condition of hyperasthesia. The patient was dismissed with a request that he call daily to have the sac cleansed and dressed.

Sept. 15. Examined surface of bone, found it here and there slightly rough, indicating that the action of the acid had not been effectual, so I repeated the application, which was followed by quite severe pain for a time until the obtundent soothed the part into quiet.

Sept. 18. I removed particles of dead tissue, the effect of first application of the acid, which were detaching, and exposed faint evidences of granular formation. A pointed instrument drawn over the bone revealed a perfectly smooth surface, excepting at the extreme lower terminus of the sac at the angle of the jaw, there was a small spot of carious surface remaining. I saturated a pellet as before, the size of a duck shot, and conveyed it on the point of the instrument until I met resistence, and left it, to add to the beautiful effects already so satisfactorily accomplished, this time giving no pain whatever. The patient now being relieved from the cause and under treatment for its effects, though so short a time had elapsed, improvement in the general constitutional conditions were decidely apparent. The greatest change of all, however, was lifting from the mind the terrible apprehension of an early anticipatied and an inevitable fate. Hope now took the place of despair. Being naturally vivacious and buoyant in spirit, the patient now gave full range and expression to his feelings and convictions, in the bright expectation of a final recovery. One of his outbursts was: "Thank heaven, the battery in my jaw is silenced, the pains are like sentry shots, only now and then, and insignificant." He was assured that repair would be slow, that time would be required for artificial assistance; more than all, the asserting and powerful forces of nature, to eliminate his heavily surcharged system from the poison and its consequences.

Sept. 28. The patient, as usual, calls daily. Has actively resumed his vocation, walking several miles daily, appetite excellent, digestive organs fully responsive, sleeps sound and refreshingly all night. Has occasional shooting pains through his body and limbs, which he designates as farewell shots. Has yet to depend upon purgatives, though light mineral waters, to keep his bowels evacuated. Every condition connected with the sac as favorable as could be expected. The pus secreted is healthly and not excessive, the surface of the bone being entirely free from the least vestage of caries—smooth as glass, and the walls of the opening as far as can be explored with the aid of a mouth mirror, are lined with granulations. The processes of repair are excusably slow, but every day there is an advance. There is entire absence of pain in that side of the face. The second molar, upon test, is free from tenderness. Altogether the progress is phenomenal.

Oct. 10. The posterior terminus of the sac is filled and the walls studded with full healthy granulations. Improvement is marked upon every feature of the case. The first glimpse of the patient as he calls from day to day, reveals the astonishing and not less pleasing change in mind and body. Reporting that although working hard every day, he feels splendid, and physically capable of almost any amount of endurance.

I will mention in this connection that the patient, through the gift by inheritance of a wonderfully strong and healthy constitution, backed by an equally pronounced and uncompromising will force, and brave and resolute heart, has unquestionably, alone, carried him through his fearfully trying ordeal of a thirty years' struggle with this hydra-headed monster of diseases. Ten thousand ordinary men would long since have succumbed, had they been subjected to the same contest and under like circumstances, proving that determination, will force, concentrated will force, is almost omnipotent over the disease ravages of the body.

Oct. 25. There remains but a small inverted cone-shaped cavity at the external opening of the recent abscess sac. I will take occasion to state that I claim no credit in the discovery of this cause, for I had a pointer. Pus had been discovered in that locality and it was plain that there was a producing cause. It was for me to find it. A word now, in reference to the tooth. When discovered as heretofore stated, three fourths or more of its grinding surface was covered with gum tissue from posterior margin forward. It may have erupted high up, from the base of the ramus, tilting the crown forward and against the second molar, producing pressure and consequent restriction in its growth, and from that cause, producing inflammation, suppuration, and after a time, absorption of the alveolus and the root, liberating it to float in the pus and act as a valve in preventing quite, or almost entirely, the escape of the secretion in the abscess. This growth of wisdom teeth is not common, yet I have seen several. Nature gives us many freaks, this may have been one in this case, or in not fully erupting, yet occupying a normal position, may be one, and as it erupted well back and near the base of the ramus, consequently, under the thicker folds of tissue, it may have grown to a point to produce these complications, and then for some cause stopped. So it was, that it never fully or normally erupted, or it would not have been so nearly covered with tissue.

It is the experience of the largest proportion of mankind to suffer more or less when erupting the wisdom teeth. We are often required to administer to, or operate for, the relief of most distressing cases. I regard them the most troublesome and dangerous teeth in the adult mouth, for the above reasons, and from the fact that they ordinarly decay while, or soon after erupting, and by the timid or indifferent, allowed to remain and become the cause of abscesses, and from their location, often during the period of inflammation and suppuration, the most serious complications are experienced.

I wish here to express a conviction entertained for many years and manifested and enforced in my practice upon all occasions, that when I could not treat an alveolar abscess successfully, arising from whatever cause, I have urged, and that at once, the removal of the cause. For the reason that I have always been satisfied that more or less of the vitiated secretion was absorbed and taken up by the blood directly, and that unquestionably the almost continual exudations from around the diseased teeth and roots, and through the fistulous openings left by collapsed abscesses, were conveyed to the stomach, and from there diffused with not less virulence and injurious results; and for another not unimportant reason by any means, the suppression of exhalations from the breath. While on this subject I will assert that I am opposed to, the practice of some, of filling the crown cavity and making, or leaving, a tap opening through the root, or plug, for the escape of foul and poisonous gases and secretions. It should be condemned. It is not creditable, it is not in consonance with the elevated stand, intelligence and advance of our profession. The maxillary sinus is not infrequently involved by diseased teeth and roots, inflammation extending through the base to the lining membrane, followed by secretion and infiltration of a sanious fluid and if neglected for any considerable time becomes a cause for grave local disturbance and serious constitutional injury. Finally, I cannot but declare, that a mouth studded with decaying and abscessed teeth contributes, without question or doubt, a prolific cause, in a greater or less degree, to pyæmia. The proof is incontestable from the case under consideration. This subject has proven cumulative, and tediously so perhaps, but regarding it as second to no other claiming our attention, I will be indulged in adding a few of the remarkable characteristic symptoms of our case, that may be noted as additionally important and pathognomonic of pyæmia. The fact is I am puzzled in deciding where all of them are apparently of equal interest and importance. However, first, a fact which has been clearly manifested in this case, but was unfortunately overlooked, but which may now be put on record, and will prove an unerring aid in diagnosing cases of similar cause. That all of the tumors were furuncular, as before stated, and every one of them, from the smallest size to the largest, contained cores. Syphilitic abscesses and ulcers do not. You will remember the condition of the hands and feet. Syphilis has no such symptoms. An eruption that quite covered the body, especially the inside of the thighs, legs and groin, and on the arms, breast and sides, that upon first appearance the papillæ were red, intensely tender, and passed to a suppurative state, when they itched intolerably, almost to frenzy, not unlike in appearance and characteristics to scabies, entirely disappearing for a considerable period before recurring again. Among the many classifications of eruption syphilis has no such one. One symptom that has prevailed almost from the first of his attack was indigestion, which has not only been the cause of serious functional distress, but grave constitutional derangement. Rarely took food with appetite, forced what he eat generally in order to maintain vitality. He never indulged the habit of drinking except an occasional glass of wine, never used tobacco. He was several times a year suddenly prostrated with paroxysms or convulsions, in which he experienced the most frightful agony, and no one organ involved more than another strange to say, but his whole body from head to foot would be held rigidly in a spasm for an hour or so, when he would loose consciousness for a few moments in a distressing nausea, recovering with excessive vomitings and purgings, and attended and followed by most excessive sweatings. Naturally, of course, leaving him utterly prostrated for several days. These spells have increased in frequency of late years and also in severity. Within the past seven months he has had five, more than ever before in the same time. You will recall he had one of his "spells," as he called them, after his first call upon me, and he stated that it was the worst of his life; the most alarming, protracted and complicated; it was the last one. A remarkable fact connected with this case is that the mind of the patient has never been affected; or long since, he says, suicide would have ended his torture. Though many times hopeless and despairing, receiving no benefit from all the skill employed, yet the mind always rallied through the powerful influence of the will, and so he kept up the contest, hoping for a finally successful issue.

Oct. 29th. The patient declared to sum up his condition altogether, that he was almost himself again; had gained fourteen pounds and is gaining every day; enjoys almost complete immunity from pain, only now and then a little stinging dart through his little finger on his left hand, and his little toe on his left foot. Was at a banquet the night before, ate a hearty supper, made a speech of half an hour, drank a few glasses of wine and felt no ill effects whatever.

Nov. 10th. About a week since a tumor commenced forming on the left hand of the patient between the bases of the metacarpal bones of the thumb and index finger, very tender, and in three or four days attaining the size of a prune. The hand is very much swollen, creating some febrile and constitutional disturbance. It now has six or seven openings discharging bloody pus, and has all the characteristics of a carbucle. He has no indications of others and is generally doing well under the circumstances. The lesion of the jaw is almost entirely closed and void of all further annoyance.

This report has unconsciously lengthened for the reason that there were so many points and phases in the case that were of so much interest and importance. The hope is that it will reflect some light to aid the various specialists and the general practitioner in making diagnoses of cases that have heretofore been perplexing and obscure as to cause, and that they will be impressed with the conviction that pyæmia from some cause, and that the oral cavity is perhaps the most prolific in producing causes, of very many more than heretofore accredited of the diseases, nerve derangements, and surely some of the gravest ills to which flesh is heir.

Nov. 17th. I will add the result and attendant symptoms of the tumor, as it may interest, up to the present. It made its appearance on the 5th inst. and culminated on the 13th. Nine openings in all formed. On the 13th the lancet was used and its contents of core and secretion removed. On the next day it had collapsed, the swelling had left the hand, and its use entirely restored and was absolutely free from tenderness. The secretion has been inconsiderable in quantity, parts healthy and healing rapidly. There was considerable distress in the region of the pyloric orifice during the time, and shooting pains radiated therefrom, strange to say, to his hand and foot, involving the head and other parts only to a limited degree. There was unquestionably a reflex action occasioned through the derangement caused by the tumor upon the hyper-sensitive diaphragm and contiguous organs, and thus a fusilade was kept up between the two points. The temporary trouble of the bowels subsided immediately after the extirpation of the core, and he is again on the rising plane of improvement. The remarkable feature will be readily observed, as to the short period between the appearance of the tumor and its culmination and the almost magic subsidence of all its complications, exemplifying most conclusively the relation between cause and effect.—*The Cincinnati Lancet and Clinic.*

